# SAGEFusionNet: An Auxiliary Supervised Graph Neural Network for Brain Age Prediction as a Neurodegenerative Biomarker

**DOI:** 10.3390/brainsci15070752

**Published:** 2025-07-15

**Authors:** Suraj Kumar, Suman Hazarika, Cota Navin Gupta

**Affiliations:** 1 Neural Engineering Lab, Department of Biosciences and Bioengineering, Indian Institute of Technology Guwahati, Guwahati 781039, India; k.suraj@iitg.ac.in; 2 Department of Radiology and Imaging, Apollo Hospitals, Guwahati 781005, India; sumanhazarika@rediffmail.com

**Keywords:** graph neural network, sMRI, anatomical graph, brain age, grey matter volume, white matter volume

## Abstract

**Background:** The ability of Graph Neural Networks (GNNs) to analyse brain structural patterns in various kinds of neurodegenerative diseases, including Parkinson’s disease (PD), has drawn a lot of interest recently. One emerging technique in this field is brain age prediction, which estimates biological age to identify ageing patterns that may serve as biomarkers for such disorders. However, a significant problem with most of the GNNs is their depth, which can lead to issues like oversmoothing and diminishing gradients. **Methods:** In this study, we propose SAGEFusionNet, a GNN architecture specifically designed to enhance brain age prediction and assess PD-related brain ageing patterns using T1-weighted structural MRI (sMRI). SAGEFusionNet learns important ROIs for brain age prediction by incorporating ROI-aware pooling at every layer to overcome the above challenges. Additionally, it incorporates multi-layer feature fusion to capture multi-scale structural information across the network hierarchy and auxiliary supervision to enhance gradient flow and feature learning at multiple depths. The dataset utilised in this study was sourced from the Alzheimer’s Disease Neuroimaging Initiative (ADNI) database. It included a total of 580 T1-weighted sMRI scans from healthy individuals. The brain sMRI scans were parcellated into 56 regions of interest (ROIs) using the LPBA40 brain atlas in CAT12. The anatomical graph was constructed based on grey matter (GM) volume features. This graph served as input to the GNN models, along with GM and white matter (WM) volume as node features. All models were trained using 5-fold cross-validation to predict brain age and subsequently tested for performance evaluation. **Results:** The proposed framework achieved a mean absolute error (MAE) of 4.24±0.38 years and a mean Pearson’s Correlation Coefficient (PCC) of 0.72±0.03 during cross-validation. We also used 215 PD patient scans from the Parkinson’s Progression Markers Initiative (PPMI) database to assess the model’s performance and validate it. The initial findings revealed that out of 215 individuals with Parkinson’s disease, 213 showed higher and 2 showed lower predicted brain ages than their actual ages, with a mean MAE of 13.36 years (95% confidence interval: 12.51–14.28). **Conclusions:** These results suggest that brain age prediction using the proposed method may provide important insights into neurodegenerative diseases.

## 1. Introduction

The structure of the human brain alters significantly over the span of a lifetime, and specific patterns of healthy brain ageing have been noticed [1,2]. These structural changes are not a medical condition but an inevitable consequence of ageing. However, these changes make it more likely for older people to develop neurodegenerative diseases and dementia [3]. Due to neurodegenerative diseases such as Alzheimer’s, Parkinson’s conditions, and schizophrenia, the human brain suffers from an unusually accelerated brain ageing process [4,5,6]. These disorders indicate an altered and sometimes accelerated brain ageing pattern in people suffering from them, as they deviate from the normal patterns of healthy brain ageing. In addition to providing information about the normal ageing process, these deviations commonly referred to as the brain age gap refer to the difference between an individual’s chronological age and brain age, which has considerable promise as a useful biomarker for detecting and investigating neurodegenerative disease, cognitive decline, and other age-related conditions [7,8,9]. The chronological age is the age of a person in years since birth, also known as actual age. Brain age, on the other hand, is determined by considering the structural and functional characteristics of the brain, which is also known as biological age [7,8]. The accurate estimation of brain age from neuroimaging data has emerged as a vital area of research. Although brain age estimation has been extensively explored in healthy populations using T1-weighted structural MRI, its application in Parkinson’s disease (PD) is still relatively limited [7,10]. In Parkinson’s disease (PD), networks that control motor, cognitive, and emotional activities are disrupted by widespread cortical and subcortical atrophy, especially in the frontal cortex, thalamus, brainstem, and basal ganglia [11,12]. According to the results of recent research on brain age in Parkinson’s disease, patients often have altered brain age compared to healthy controls [13,14].

Structural magnetic resonance imaging (sMRI) is one of the several neuroimaging modalities that offer comprehensive anatomical details, making it an effective tool for investigating the structure of the brain [15,16] and how it relates to age. Classical techniques for predicting brain age frequently use machine learning algorithms using features derived from sMRI data [17,18,19]. Despite their good performance, these methods are unable to capture the intricate pairwise relational information between various brain regions. This issue can be addressed by Graph Neural Networks (GNNs), which operate directly on graph-structured data. The human brain can be naturally depicted as a graph, representing a complex network of interconnected brain areas. In a study, different brain regions are represented as nodes, and the physical connections between regions are represented as edges, forming a graph [20]. In recent years, the analysis of graphs using machine learning has garnered significant attention among researchers due to the remarkable expressive capabilities offered by graphs.

Graph Neural Networks (GNNs) are deep learning-based methods that extend existing deep learning techniques to non-Euclidean data by leveraging the graph structure of data to simplify learning tasks effectively [21]. GNNs have shown significant promise in processing graph-structured data by leveraging the underlying relationships within graphs [22]. Their strong performance across various tasks has made them increasingly popular in various domains. There are some studies where GNNs have been successfully applied to sMRI data, including age and gender prediction using GCNs on the cortical surface area [23], and the examination of performance disparities in brain age prediction using multi-volumetric sMRI features [24]. Moreover, semi-supervised node prediction has been performed using population graphs [25], and in a comparative examination of various message-passing and pooling methods, ROI-aware pooling showed significant improvements [26]. Most GNNs employ a sequential neighbourhood aggregation approach to learn a node’s representation vector, often referred to as node embedding, during the training process. This approach involves utilising a first-order graph filtering operation to aggregate representation vectors from a node’s immediate one-hop neighbours. The term “first-order” signifies that this operation considers only direct connections or edges between nodes, usually without considering higher-order connections or paths. These aggregated vectors are then used to compute the node’s representation vector for each iteration, forming a GNN layer [27]. Many common variants of Graph Neural Networks (GNNs) have been developed based on different choices of sequential neighbourhood aggregation strategies. These variants include Graph Convolutional Networks (GCNs) [22], Graph Sample and Aggregated Layers (GraphSAGEs) [28], Graph Attention Networks (GATs) [29], and Graph Isomorphism Networks (GINs) [30]. Each of these approaches employs distinct methods to aggregate features from a node’s neighbours in the graph. These techniques play a crucial role in determining the trajectory of the learning process and the expressiveness of the final GNN model.

Through cascading multiple GNN layers, a variety of tasks, such as node classification [22,31], edge classification [32], graph classification [33], link prediction [34], and various complex problems including those found in social networks [35], have been performed. This sequential cascading, along with non-linear activations, enables Graph Neural Networks (GNNs) to aggregate features beyond one-hop neighbours, leading to enhanced generalisation. For instance, when two such layers are cascaded along with non-linearity, information from two-hops-away neighbours is aggregated. Similarly, cascading three, four, or more layers aggregates features from three-, four-, or more-hops-away neighbours, respectively, thus improving model performance. However, due to successive aggregations, the node embeddings converge towards a non-informative state [36]. When it comes to mean aggregation, node embeddings become increasingly similar and eventually indistinguishable; this phenomenon is called oversmoothing [36,37]. This issue, along with others like the vanishing gradient problem, makes sequential aggregation-based GNN models susceptible to performance degradation due to the cascading of multiple layers and the network growing deeper [22,37,38]. Geometric deep learning research on oversmoothing is quite active, and it would be outside the purview of this article to provide a comprehensive list of works. Research has mostly concentrated on new architectures to alleviate it, like randomly deleting connections and residual techniques [39,40], using regularisations [41], as well as ingenious normalisation [42,43]. These challenges pose significant obstacles to designing deeper GNN models and result in the loss of important information available beyond one-hop neighbours. The motivation of this study is to develop a GNN-based framework that enables the design of deeper models without encountering these issues.

The aim of this study is to develop a novel and enhanced GNN-based framework for brain age prediction. This integrates the expressive capabilities of GNNs at the ROI level with T1-weighted structural MRI (sMRI) data. The suggested approach attempts to represent the brain as a graph in order to capture its rich topological structure. The techniques used in our model, such as node-level pooling [26,44] and multi-depth supervision [45], have already been investigated in other studies. The novel aspect of our work is the strategic integration of these techniques within the proposed SAGEFusionNet architecture, which combines them in a unified framework. Our model incorporates ROI-aware pooling at each layer for identifying important ROIs in brain age prediction, multi-layer feature fusion to capture multi-scale structural information, and auxiliary supervision to enhance feature learning across different network depths. This integration enables the model to enhance its performance by mitigating oversmoothing, enhancing gradient flow, and improving representational diversity, as supported by the comparative results and Dirichlet energy analysis. Additionally, in order to confirm the clinical applicability of our approach, we tested the proposed model on Parkinson’s disease (PD) datasets. While some of these techniques have been used individually in prior studies, to the best of our knowledge, this particular integration strategy and its application to brain age prediction in Parkinson’s Disease using GNNs have not been previously reported. This manuscript is divided into the following sections and subsections: Section 2 provides an outline of the proposed approach, including information about the dataset, preprocessing, construction of the anatomical network, model description, training, and testing. The results and the discussion are presented in Section 3 and Section 4. The conclusion of the proposed study is finally described in Section 5.

## 2. Materials and Methods

### 2.1. Dataset Description

The dataset utilised in this study was sourced from the Alzheimer’s Disease Neuroimaging Initiative (ADNI) database (https://adni.loni.usc.edu/, accessed on 11 January 2024), a large public repository that offers neuroimaging, neuropsychological, clinical, and genetic data aimed at tracking the development of Alzheimer’s disease dementia. The ADNI datasets are multisite datasets, containing data obtained from multiple MRI scanners and locations across various research sites. Our study incorporated a total of 580 T1-weighted MRI scans obtained from healthy subjects, comprising 233 male and 347 female scans. The subjects included in our study were carefully selected to ensure age and gender matching, with ages ranging between 51 and 95 years. The average age of male subjects was 74.99 ± 7.08 years, while that of female subjects was 72.70 ± 6.94 years. As per the ADNI Data Manual, these subjects were chosen based on the following criteria: no significant memory complaints beyond age-related norms; normal memory function confirmed by Logical Memory II scores meeting education-specific cutoffs (≥9 for 16+ years, ≥5 for 8–15 years, ≥3 for 0–7 years of education); Mini-Mental State Exam score between 24 and 30; Clinical Dementia Rating of 0 with a Memory Box score of 0; and overall cognitive normality with no notable impairments in cognition or daily functioning. Another 215 Parkinson’s disease (PD) patient scans were used in this study, obtained from the Parkinson’s Progression Markers Initiative (PPMI) database (www.ppmi-info.org/access-data-specimens/download-data, accessed on 30 July 2023) to assess brain ageing patterns related to PD. Among these, 129 were male and 88 were female, with an average age of 58.47 ± 7.76 years. As per the PPMI Clinical Protocol Manual, these PD patients were chosen based on the following criteria: if they were at least 30 years old, had a clinical diagnosis of PD, and displayed at least two motor symptoms (such as stiffness, bradykinesia, or resting tremor), with either bradykinesia or resting tremor being necessary. Prior to receiving a DaTscanTM injection, participants had to meet UPSIT requirements, give informed consent, pause specific drugs before SPECT imaging, and, if they were female, test negative for pregnancy.

### 2.2. Preprocessing

**Figure 1:**
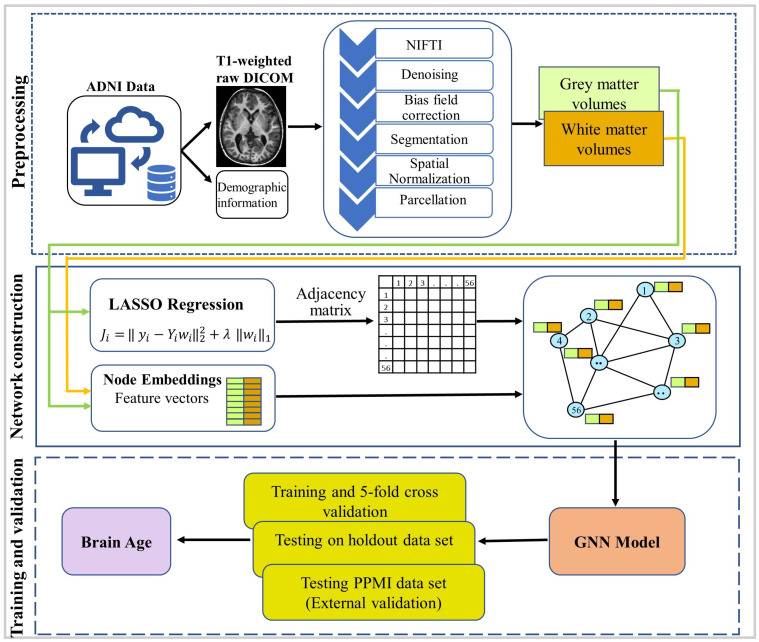
Graphical abstract of proposed framework for brain age prediction.

The raw T1-weighted structural sMRI images obtained from the Alzheimer’s Disease Neuroimaging Initiative (ADNI) were initially in Digital Imaging and Communications in Medicine (DICOM) format. These images were converted to Neuroimaging Informatics Technology Initiative (NIfTI) format using the dcm2nii tool from MRIcron (Version:v1.0.2019092) [46]. Subsequently, these scans underwent preprocessing with the Computational Anatomy Toolbox (CAT12, Version: vCAT12.8.2) [47], within Statistical Parametric Mapping (SPM12) (https://www.fil.ion.ucl.ac.uk/spm/software/spm12/, accessed on 8 August 2023), using MATLAB 2022a [48]. Major preprocessing steps are depicted in Figure 1; the NIfTI scans were denoised using a spatial-adaptive Non-Local Means (SANLM) denoising filter, which enhances image quality by removing noise while preserving edges. Bias field correction was then performed to mitigate intensity inhomogeneities by minimising the effects of the bias field. Following this, the scans were segmented using tissue probability maps, dividing them into three primary tissues: grey matter (GM), white matter (WM), and cerebrospinal fluid (CSF). The segmented scans were then spatially normalised to Montreal Neurological Institute (MNI152) standard space. Subsequent to normalisation, the images were further processed using the LONI Probabilistic Brain Atlas (LPBA40) for parcellation, which divided the brain into 56 regions of interest (ROIs) [49]. Each ROI represents a specific anatomical area within the brain. The regional grey matter $GM_{\mathrm{MN}}$ and white matter $WM_{\mathrm{MN}}$ volumes for each subject were extracted, where $GM_{\mathrm{MN}}$ denotes the grey matter volume and $WM_{\mathrm{MN}}$ denotes the white matter volume of the $N^{\mathrm{th}}$ ROI for the $M^{\mathrm{th}}$ subject. These volumes of each ROI were normalised by the subject’s intracranial volume to account for individual head size differences. Subsequently, the mean and standard deviation of each normalised ROI volume were computed across subjects and used to standardise the corresponding ROI volumes, resulting in features with zero mean and unit variance. Therefore, for *M* subjects, each having *N* ROIs, we obtained subject vs. ROI volume matrices $X_{\mathrm{GM}}$ and $X_{\mathrm{WM}}$, as shown in Equations (1) and (2), each with dimensions M×N. Only the $X_{\mathrm{GM}}$ volume feature was used for further construction of the anatomical network.

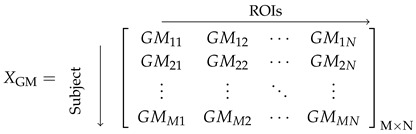
(1)

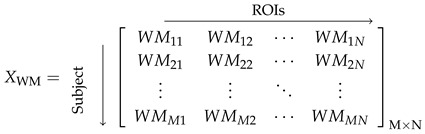
(2)

### 2.3. Construction of Anatomical Network

A graph is a mathematical structure consisting of a set of nodes (V) and edges (E), represented as G=(V,E). Nodes denote specific entities or points, while edges indicate the relationships or connections between them. An edge (u,v) in graph *G* illustrates a connection between node u∈V and node v∈V. In this study, we constructed an anatomical network using a correlation-based method with LASSO optimisation, a graph learning technique derived from the literature of graph signal processing (GSP) [50,51]. The network’s nodes represent various brain regions of interest (ROIs), and the edges show the associations of regional grey matter $GM_{\mathrm{MN}}$ volume among these ROIs obtained from structural MRI (sMRI) data [26]. The specific dependencies depicted by the edges can vary based on the graph construction method and the inherent constraints of the optimisation problem. An anatomical network was constructed from data of *M* subjects, each having *N* brain ROI volumes, by applying LASSO optimisation [26,51].

For an arbitrary node *i*, the edge weights $w_{i}$ from the node *i* to all other nodes m=1,2,…,N−1, ∀m≠i, can be derived by minimising the optimisation function $J_{i}$. In matrix form, it is given by

(3) $$J_{i}=\left|\right|y_{i}-Y_{i}w_{i}{\left|\right|}_{2}^{2}+\lambda ||w_{i}{\left|\right|}_{1}$$

where $w_{i}={\left[w_{i2},w_{i3},w_{i4},\ldots ,w_{im}\right]}^{T}$, i=1,2,3,…,N (ROIs), and m=1,2,…,N−1, ∀m≠i. $y_{i}$ is the *i*-th column of $X_{GM}$, which represents the *i*-th ROI across all the subjects, $Y_{i}$ is the $X_{GM}$ matrix after removing the *i*-th column, and λ is the sparsity parameter. The first term encourages correlation among connected nodes, whereas the second term encourages sparsity. A sparser graph results from a larger value of λ, and its optimal value λ=0.06 was selected based on multiple experiments given in Table 1.

The optimisation problem mentioned above needs to be solved individually for every node. This process produces a set of edge weights or coefficients, denoted as $w_{i}$, for each node *i*, resulting in *N* sets of weights corresponding to *N* nodes. In order to maintain dimensional consistency, a zero is padded at the *i*-th position of $w_{i}$ since the *i*-th ROI is eliminated during regression. These sets of weights are then concatenated together to form the matrix *W*, which contains all the edge weights for the entire network. However, this process does not guarantee that *W* will be a symmetric matrix. An adjacency matrix, by definition, is symmetric. To confirm symmetry, the matrix *W* is multiplied by its transpose $W^{T}$, and then the square root is taken to obtain the adjacency matrix depicted in Equation (4). The size of the obtained adjacency matrix (A) will be N×N.

(4) $$A_{N\times N}=\sqrt{W\cdot W^{T}}$$

**Figure 2:**
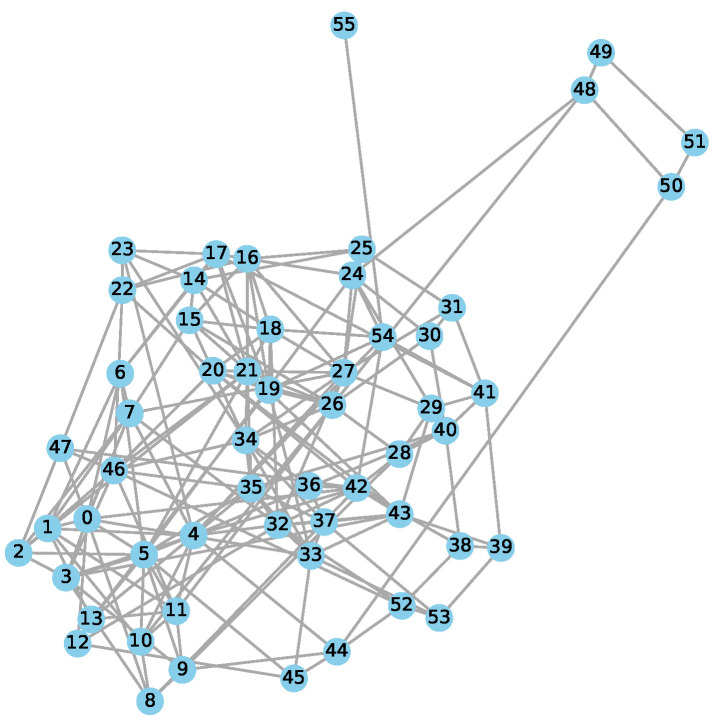
Anatomical brain network constructed using grey matter.

Using the aforementioned technique, an unweighted anatomical graph is constructed, where *N* represents the number of nodes in the network depicted in Figure 2. The nodes of these graphs are initialised with regional $GM_{\mathrm{MN}}$ and $WM_{\mathrm{MN}}$ volumes features instead of structural or location-based attributes [52].

### 2.4. Model Description

**Figure 3:**
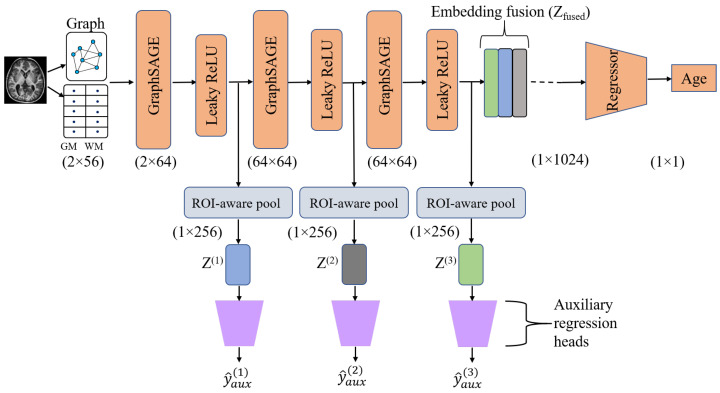
Architecture of SAGEFusionNet model.

The GNN model proposed in this study consists of multiple layers working together to generate the final output depicted in Figure 3. The anatomical graph generated from sMRI using Equations (3) and (4) was utilised as the input for the proposed architecture. Every node in this graph has two features representing the regional grey matter $GM_{\mathrm{MN}}$ and white matter $WM_{\mathrm{MN}}$ volumes of a specific brain region extracted from sMRI. These features are encapsulated within the feature matrix $X\in R^{N\times F}$, where N symbolises the total brain ROIs for each subject and F represents the number of features associated with each node or ROI. The initial layer comprises the Graph Sample and Aggregation (GraphSAGE) layer, which is a fundamental element of the proposed model introduced by William L. Hamilton [28]. GraphSAGE is a flexible inductive framework designed for generating node embeddings by sampling and aggregating features from a node’s local neighbourhood. Instead of considering the entire neighbourhood, GraphSAGE uniformly samples a fixed-size subset of neighbours for aggregation. The function AGG(·) represents the aggregation function, and GraphSAGE proposes three types of aggregators: mean aggregator, LSTM aggregator, and pooling aggregator. In the proposed model, we used a mean aggregator in GraphSAGE that can be considered an inductive version of Graph Convolutional Networks (GCNs). In contrast, the LSTM aggregator is not permutation-invariant, as it relies on a specified order of nodes for aggregation. We selected the GraphSAGE layer for our approach because it offers superior inductive performance and scalability.

Additionally, as shown in Table 2, the GraphSAGE-based model performed better in our study than other standard GNN architectures under the same experimental setting. The main goal of the GraphSAGE layer is to combine node features with graph structural features to update node representations. This involves aggregating features from neighbouring nodes along with the node’s own features. If there are *r* GraphSAGE layers [28], then the node update equation for node *v* at layer *r* is given by Equation (5):

(5) $$x_{v}^{\left(r\right)}=\sigma \left(W_{r}\left[\mathrm{AGG}\left(\left\{x_{u}^{\left(r-1\right)},\forall u\in N\left(v\right)\right\}\right),x_{v}^{\left(r-1\right)}\right]\right)$$

In Equation (5), $W_{r}$ represents a learnable weight matrix, $x_{v}^{\left(r\right)}$ denotes the updated features of node *v* at the *r*-th layer, σ signifies the non-linear activation, specifically Leaky ReLU, N(v) denotes the neighbours of node *v*, and $x_{u}^{\left(r-1\right)}$ denotes the node embedding of neighbouring node *u* at the (r−1)-th layer.

The output of the first GraphSAGE layer was passed through a non-linear activation function called Leaky ReLU. The purpose of using Leaky ReLU was to enable the learning of more complex relationships and patterns in the data and to overcome the issue of vanishing gradients. We standardised the feature to have a zero mean and unit variance. As a result, some of the features became negative, but these negative values are still considered by the Leaky ReLU activation function; this is an additional advantage of using Leaky ReLU, unlike the standard ReLU activation function, which discards all negative values and outputs only positive values. The output of this Leaky ReLU was the aggregated embeddings of one-hop away neighbours, which were passed to the next GraphSAGE layer and ROI-aware pooling layer.

#### ROI-Aware Pooling

It is a multi-head weighted pooling mechanism where each head learns a soft ROI attention over nodes and aggregates node embeddings into a graph-level representation [26]. Consider the node feature matrix $X\in R^{N\times F}$, where *F* is the feature dimension and *N* is the number of nodes. $W\in R^{N\times H}$ is a learnable weight matrix, where *H* denotes the number of attention heads. The attention score vector for a single head across all nodes is represented by each column of *W*. A softmax function is applied to produce a normalised attention score vector:

(6) $$A=\mathrm{softmax}\left(W\right)\in R^{N\times H}$$

The graph-level embedding for each attention head *h* is given by $z_{h}$:

(7) $$z_{h}=\sum_{i=1}^{N}A_{ih}\cdot x_{i}$$

Lastly, the outputs from each attention head are concatenated to create the overall graph-level representation at layer *l*:

(8) $$Z^{\left(l\right)}=[z_{1}∥z_{2}∥\ldots ∥z_{H}]\in R^{H\cdot F}$$

The output of the first ROI-aware global pooling is given as $Z^{\left(1\right)}$. The proposed model consists of four cascaded GraphSAGE layers along with Leaky ReLU activations. Similarly, the second, third, and fourth GraphSAGE layers’ outputs passed through subsequent ROI-aware global pooling, which produces graph-level embeddings $Z^{\left(2\right)},Z^{\left(3\right)}$, and $Z^{\left(4\right)}$ containing aggregated features from the second-, third-, and fourth-hops-away neighbouring nodes. The graph-level outputs from each layer are

$$\mathrm{outputs}=\left(Z^{\left(1\right)},Z^{\left(2\right)},\ldots ,Z^{\left(4\right)}\right)$$

These ROI-aware pooling output embeddings $Z^{\left(1\right)}$, $Z^{\left(2\right)}$, …, $Z^{\left(r\right)}$ after each GraphSAGE layer are passed to the corresponding auxiliary regression head, which produces the auxiliary prediction at each layer, respectively: ${\hat{y}}_{\mathrm{aux}}^{\left(1\right)}$, ${\hat{y}}_{\mathrm{aux}}^{\left(2\right)}$, and so on, until ${\hat{y}}_{\mathrm{aux}}^{\left(r\right)}$, which are shown in Figure 3. Using these auxiliary predictions, the auxiliary losses are calculated at each layer. After processing all layers, the model fuses the outputs from each ROI-aware global pooling together. A variety of approaches, including mean, max, sum, weighted sum, attention-based mechanisms, and concatenation, can be used to accomplish this fusion, and their performance is presented in Table 3. In the case of concatenation, it can be given by

(9) $$Z_{\mathrm{fused}}=\left[Z^{\left(1\right)}∥Z^{\left(2\right)}∥\ldots ∥Z^{\left(r\right)}\right]$$

Subsequently, this fused embedding was passed to the regressor layer to produce the predicted value of brain age. The regressor layer is a multi-layer perceptron (MLP) comprising a fully connected layer (FC), a Leaky ReLU layer, and another FC layer with a single neuron responsible for producing the predicted brain age. Since our proposed model incorporates GraphSAGE layers and a fusion mechanism, we refer to it as SAGEFusionNet. All the steps of the proposed model are summarised in Algorithm 1. As part of this study, baseline models including FCNN, GCN, GraphSAGE, GIN, and GAT were incorporated for comparison purposes.
**Algorithm 1** SAGEFusionNet model algorithm**Required inputs:** Graph G=(V,E) (edge_index), initial node features $X\in R^{N\times F}$, number of GraphSAGE layers *r*, dropout rate ρ, regularisation hyperparameter α**Output:** Auxiliary predicted value at *i*-th layer ${\hat{y}}_{\mathrm{aux}}^{\left(i\right)}$, final predicted value $\hat{y}$Initialise auxiliary regression head ${\mathrm{Reghead}}_{\mathrm{aux}}^{\left(i\right)}$ for prediction at *i*-th layerInitialise lists: pooled_outputs←[ ], aux_losses←[ ]**for** i=1 to *r* **do**    $X\leftarrow \mathrm{LeakyReLU}({\mathrm{SAGEConv}}_{i}\left(X,\mathrm{edge}\_\mathrm{index}\right))$    X←Dropout(X,p=ρ)    $X_{\mathrm{pooled}}\leftarrow \mathrm{ROIAwareGP}\left(X,\mathrm{batch}\right)$    Append $X_{\mathrm{pooled}}$ to pooled_outputs    ${\hat{y}}_{\mathrm{aux}}^{\left(i\right)}\leftarrow {\mathrm{Reghead}}_{\mathrm{aux}}^{\left(i\right)}\left(X_{\mathrm{pooled}}\right)$    $L_{\mathrm{aux}}^{i}\leftarrow \mathrm{MSE}\left({\hat{y}}_{\mathrm{aux}}^{\left(i\right)},y\right)$    Append $L_{\mathrm{aux}}^{i}$ to aux_losses**end for**$\;Z_{\mathrm{fused}}\leftarrow \mathrm{Concat}\left(\mathrm{pooled}\_\mathrm{outputs}\right)$$\hat{y}\leftarrow \mathrm{Regressor}\left(Z_{\mathrm{fused}}\right)$$L_{\mathrm{aux}}\leftarrow \frac{1}{r}\sum_{i=1}^{r}L_{\mathrm{aux}}^{i}$$L_{\mathrm{hybrid}}\leftarrow \frac{1}{n}\sum_{i=1}^{n}{\left({\hat{y}}_{i}-y_{i}\right)}^{2}+\alpha \cdot L_{\mathrm{aux}}$**return** $\hat{y}$

### 2.5. Training and Testing

In our study, a careful approach was taken to train the model effectively. All models in this study were trained using Google Colab, (https://colab.research.google.com/, accessed on 1 May 2025) which offers a virtual machine (VM) environment equipped with 12.67 GB of RAM and 107.72 GB of available disk space. The experiments were conducted using Python 3.11, leveraging the PyTorch framework and the PyTorch Geometric library for implementing Graph Neural Network (GNN) models. Initially, the dataset was split into two parts: one for training the models and the other for testing their performance, with an 80:20 ratio, respectively. To ensure the reliability and generalisation of the models, a 5-fold cross-validation technique was employed, involving repeated training and validation with different data combinations. The models were trained using mini-batches of 16 samples to enhance computational efficiency. An adaptive learning rate strategy was implemented, starting with an initial learning rate of 0.01 to adjust the learning rate during training dynamically. To prevent overfitting, a dropout rate of 0.15 was applied. A hybrid loss function given in Equation (10) was employed during training to incorporate the auxiliary loss. Several values of the hyperparameter α were evaluated, and our model achieved optimal performance at α=0.30. To further optimise the learning process, an Adam optimiser was employed to accelerate convergence and stabilise the training dynamics.

Loss Function

The hybrid loss function used in our study is a combination of two loss functions, the Mean Squared Error (MSE) loss and an auxiliary loss function, to ensure robustness in capturing differences between predicted brain age and actual age. It is given by the following Equation (10):

(10) $$L_{\mathrm{hybrid}}=\underset{L_{\mathrm{main}}}{\underset{︸}{\frac{1}{n}\sum_{i=1}^{n}{\left({\hat{y}}_{i}-y_{i}\right)}^{2}}}+\alpha \cdot \underset{L_{\mathrm{aux}}}{\underset{︸}{\frac{1}{r}\sum_{l=1}^{r}\mathrm{MSE}\left({\hat{y}}_{\mathrm{aux}}^{\left(l\right)},y\right)}}$$

where *n* represents the number of subjects, $y_{i}$ denotes the actual age of the *i*-th subject, and ${\hat{y}}_{i}$ refers to the predicted brain age for the *i*-th subject. The variable *r* denotes the number of GraphSAGE layers in the model, α is a regularisation hyperparameter, and ${\hat{y}}_{\mathrm{aux}}^{\left(l\right)}$ denotes the predicted value at the $l^{\mathrm{th}}$ layer. The importance of the auxiliary loss in the hybrid loss function is controlled by the hyperparameter α. The main loss supervises the final prediction derived from fused ROI-aware representations across all GNN layers, while each layer receives intermediate supervision from the auxiliary loss.

#### Performance Metrics

To evaluate the efficacy of the models, the mean absolute error (MAE) and the Pearson Correlation Coefficient (PCC) were employed as metrics to assess how well the predicted age of the brain aligns with the actual age of the subjects. Additionally, the Dirichlet energy given in Equation (11) was used to analyse the oversmoothing of the models:

- Dirichlet energy:
  It is one of the metrics to measure the oversmoothing in deep GNN on graph-structured data [36,53]. The normalised version of the Dirichlet energy at the $l^{\mathrm{th}}$ GNN layer is given in the following Equation (11):
  (11) $$E\left(x^{l}\right)=\sum_{i\in V}\sum_{j\in N_{i}}{∥\frac{x_{i}^{l}}{\sqrt{1+d_{i}}}-\frac{x_{j}^{l}}{\sqrt{1+d_{j}}}∥}_{2}^{2}$$
  where V is the set of all nodes and $N_{i}$ is the set of neighbours of node *i*. The terms $x_{i}^{l}$ and $x_{j}^{l}$ represent the features of nodes *i* and *j* at the $l^{\mathrm{th}}$ layer, respectively. The terms $d_{i}$ and $d_{j}$ denote the degrees of nodes *i* and *j*, respectively. Finally, ${∥\cdot ∥}_{2}^{2}$ denotes the squared $\ell_{2}$-norm.

## 3. Results

**Figure 4:**
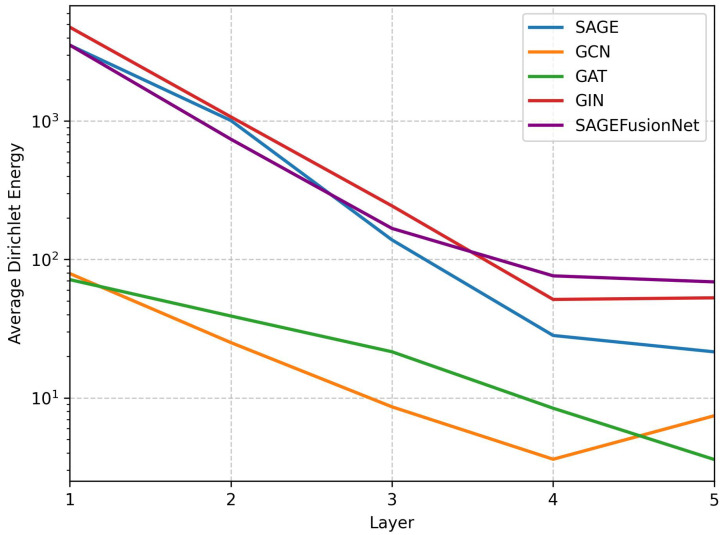
Oversmoothing analysis of various GNNs at different depths.

The performance of the proposed SAGEFusionNet model with different numbers of GraphSAGE layers is shown in Table 4. The Pearson Correlation Coefficient (PCC) and mean absolute error (MAE) were computed when the number of layers varied from two to six. Four layers achieved the most promising results, with an MAE of 4.24±0.38 and a PCC of 0.72±0.03. This finding shows that while deeper configurations (five and six layers) begin to experience a reduction in performance, moderate depth promotes optimal learning. Table 3 reports the impact of various feature fusion strategies on model performance. A number of techniques were investigated to aggregate the ROI-aware pooled representations across GNN layers, including mean, max, sum, weighted sum, attention, and concatenation. The concatenation approach performed better than the others. This finding suggests that concatenation, rather than averaging or summing the embeddings into a single vector, better aids prediction by maintaining the distinctiveness of each layer’s representation. Figure 4 shows the variance of Dirichlet energy across different depths of GNN layers for all other models in order to assess the oversmoothing behaviour across GNN models. Evidence of similar node embeddings can be seen in models like GCN, GAT, GIN, and GraphSAGE, where Dirichlet energy sharply declines with depth. On the other hand, SAGEFusionNet continuously sustains greater Dirichlet energy levels, indicating a more robust feature diversity preservation. This provides empirical evidence that our model uses ROI-aware pooling and auxiliary supervision at each layer to reduce oversmoothing.

The proposed framework is compared with different baselines, including FCNN, GCN, GraphSAGE, GAT, and GIN, in Table 2. SAGEFusionNet produced the most promising results out of all the other approaches, with an MAE of 4.24±0.38 and PCC of 0.72±0.03. The significance of structural modelling can be seen by the FCNN model, which performed the worst, with MAE = 6.16±0.99, PCC = 0.54±0.07. SAGEFusionNet specifically solves the oversmoothing and less efficient gradient propagation in deeper layers that hinder most of the GNN models, despite their reasonable performance.

**Figure 5:**
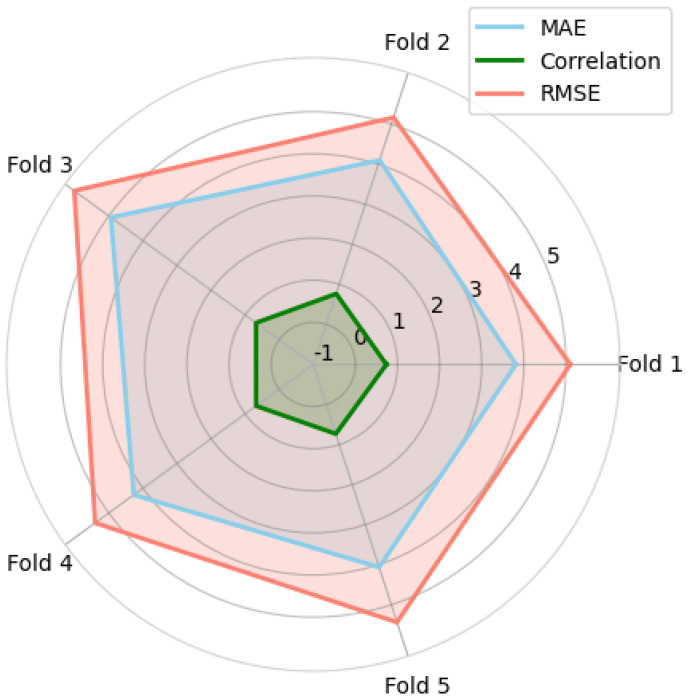
Spider plot of different matrices during 5-fold cross-validation.

**Figure 6:**
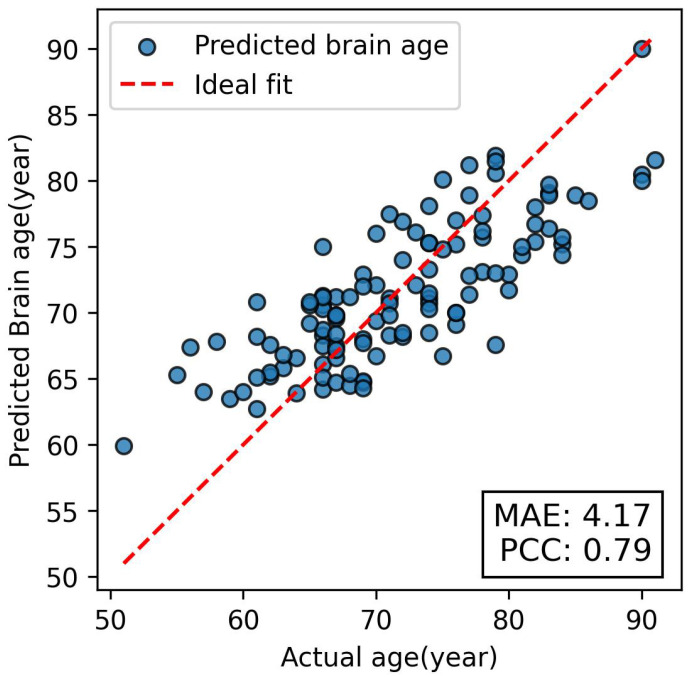
Scatter plot of actual age vs. predicted age.

A spider plot of the MAE, PCC, and RMSE metrics over five cross-validation folds is displayed within Figure 5. SAGEFusionNet’s excellent generalisation capacity is demonstrated by the consistent performance across all folds. The model’s stability and absence of overfitting on particular data partitions are highlighted by this consistent behaviour. A scatter plot of the predicted versus actual brain age values for the holdout test set is shown in Figure 6. High correlation with the ground truth is confirmed by the predictions’ near alignment with the diagonal identity line. With an MAE of 4.17 and a PCC of 0.79, the test results demonstrate the model’s strong generalisation to unseen holdout datasets and its ability to accurately capture the structural patterns of the brain, corresponding to brain age. To confirm its efficacy, we further validated the proposed SAGEFusionNet model on an external dataset comprising 215 PD patient scans from the Parkinson’s Progression Markers Initiative (PPMI) database. According to preliminary results, predicted brain ages were higher than actual ages in 213 out of 215 individuals with Parkinson’s disease and lower in only 2, with a mean absolute error (MAE) of 13.36 years (95% CI: 12.51–14.28), indicating stable predictive performance. These results appear to align with previous investigations that reported that people with Parkinson’s disease (PD) experience accelerated brain ageing [5].

## 4. Discussion

The efficacy of the proposed SAGEFusionNet model in brain age prediction tasks is clearly demonstrated by the experimental findings. Numerous modifications to GNN architecture led to enhanced brain age prediction. The incorporation of ROI-aware global pooling at every GraphSAGE layer performs a very important function. The ROI-aware pooling focuses more on important brain ROIs for brain age prediction, in contrast to traditional global pooling. Second, extra supervision at each GraphSAGE layer also helps to reduce two basic issues with deep GNNs, oversmoothing and vanishing gradients, while accelerating convergence. As we go deeper, SAGEFusionNet sustains considerably higher Dirichlet energy levels throughout layers than baseline models, which is verified by the theoretical analysis and practical observations in Figure 4. This suggests improved discriminative power and feature variance preservation at the deeper layer.

Third, to combine embeddings from multiple GraphSAGE layers, the fusion of multi-layer ROI-aware embeddings was investigated, employing a variety of approaches, such as mean, max, sum, attention, and concatenation. Table 3 demonstrates how this fusion process captures discriminative meaningful features from various network depths. It is found that models that utilised attention or concatenation fusion approaches performed better than those that employed a simple aggregation approach. These results highlight the importance of maintaining multi-scale representations to improve generalisation and reduce the effect of oversmoothing in deep GNNs. Figure 5 provides consistent findings throughout all five folds, and Figure 6 shows a strong correlation between predicted and actual brain ages, further confirming the method’s efficacy and robustness. Lastly, we tested the model using the Parkinson’s disease (PD) dataset and found that the predicted brain ages for most patients were greater than their actual ages. Our initial findings show that the MAE for healthy controls was 4.24±0.38 years, while for PD subjects it was 13.36 years. This reveals that the brain age gap in PD subjects is larger relative to their chronological age compared to healthy controls. This finding is clinically relevant because it supports the various theories that neurodegenerative diseases cause accelerated brain ageing. Structural changes associated with Parkinson’s disease are effectively captured by our model by encoding pairwise relationships between ROIs. The ability to track changes in brain structure implies that brain age estimation frameworks such as SAGEFusionNet could offer a new way to monitor the course of illness and evaluate the effectiveness of therapy. Finally, the combination of ROI-aware pooling, auxiliary regression heads, and multi-layer fusion produces a model that outperforms baseline GNNs in terms of performance, learning efficiency and stability. Together, these elements allow the model to move beyond some of the inherent drawbacks of deep GNNs. The current study shows that SAGEFusionNet can achieve excellent brain age prediction performance using only single-modality structural MRI data, reducing complexity. To the best of our knowledge, SAGEFusionNet is the first framework that encodes both global and local structural brain features acquired from grey matter and white matter volumes by utilising layer-wise ROI-aware pooling, auxiliary supervision, and multi-layer feature fusion.

Pina et al. [26] used ROI-aware pooling after the last GNN layer to learn important ROIs and transform node-level embeddings into a graph-level representation. While this method is effective, its output solely depends on the representation of the last GNN layer. Hierarchical graph pooling with structure learning [54] performs top-k node pooling at each layer, which preserves a subset of informative nodes and forms a smaller induced subgraph, while structure learning subsequently reconstructs the graph structure for this pooled subgraph. DiffPool [44] performs hierarchical graph pooling by learning a soft cluster assignment from node embeddings to coarsen the graph structure across layers, where the coarsened graph serves as input to the next GNN layer. Numerous studies have looked into feature fusion from various dimensions. For example, JK-Nets [55] fuses node-level embeddings from each GNN layer using concatenation or max-pooling, demonstrating the benefits of combining representations from multiple depths. DeepGCNs [45] incorporate residual/dense connections to pass earlier layer outputs into deeper layers while simultaneously fusing features from various depths. GCNII [40] advances this idea through initial residual connections and identity mapping, performing implicit fusion across node embeddings within each layer. However, these methods do not perform explicit graph-level feature fusion across depths. In contrast, our method applies ROI-aware pooling at each GraphSAGE layer to learn important ROIs for age prediction without altering the original graph topology. The resulting graph-level representations from different network depths are fused to capture multi-scale structural information across the network hierarchy. Additionally, this framework is supported by auxiliary supervision, enabling more expressive and hierarchical representation learning, making the model more task-adaptive.

The latest studies in brain network modelling have shown the benefits of combining multimodal data, including MRI, DTI, fMRI, and EEG, using graph-based learning methods like GNNs and Transformers. These methods have facilitated enhancement in both predictive accuracy and interpretability by capturing the association between structural and functional connectivity [56,57,58,59]. Furthermore, edge-centric models such as Edge-Boosted Graph Learning [60] provide a new perspective to conventional node-based techniques through constructing edge functional connectivity (eFC) from time series data and employing a co-embedding technique to jointly learn from node and edge features. While our current work focuses solely on structural MRI, we recognise the potential of expanding SAGEFusionNet to integrate additional data types, such as DTI for white matter tractography or fMRI for capturing functional coupling. SAGEFusionNet does not employ energy-based models to simulate brain state transitions; it uses Dirichlet energy as an analytical metric to quantify oversmoothing across network layers. This usage is conceptually aligned with energy-based frameworks where, for example, the brain selectively reallocates network energy across tasks and networks [61], energy landscape analysis has been used to characterise brain state dynamics and transitions in Alzheimer’s disease [62], and lattice field theory has been applied to describe neural activity in terms of potential and kinetic energy [63]. Similarly, physics-inspired GNNs have been used to solve combinatorial optimisation problems [64] and to estimate the haemodynamic variables of the human brain [65], underscoring the value of integrating physical priors into modelling. These models offer rigorous, biophysically grounded formulations. Integrating them could enhance interpretability and robustness. However, the age range of the training dataset (51 to 95 years) is a constraint of the current work that may limit the model’s potential to be applied to larger populations. In particular, SAGEFusionNet might underestimate brain age for people over 95 and overestimate it for people under 51. To improve the model’s resilience and suitability for a greater variety of clinical situations, future research will concentrate on validating it on bigger, more age-diverse cohorts. Additionally, including environmental and lifestyle factors through real-world testbeds such as towns, neighbourhoods, and clinics to consider their impact on brain ageing [66] may enhance the model’s ability to capture the wide contributors of brain ageing and improve generalisability.

## 5. Conclusions

In this work, we introduced a novel Graph Neural Network framework called SAGEFusionNet that uses T1-weighted structural MRI data to predict brain age. Key issues with deep GNNs, such as oversmoothing and vanishing gradients, are addressed in the proposed model by combining ROI-aware global pooling, layer-wise auxiliary supervision, and multi-layer feature fusion. Additionally, based on clinical evaluation of PD patient data from the PPMI dataset, SAGEFusionNet consistently predicts brain ages that are greater than chronological ages, indicating accelerated ageing trends in PD patients. This demonstrates the therapeutic significance of the model and its potential as a non-invasive biomarker for neurodegenerative illness. This approach may offer a novel way to assess treatment effectiveness by comparing predicted brain age to actual age and monitoring changes in the brain age gap following medication. It might also improve knowledge of disease progression and how effective treatments are. This approach for brain age prediction has the potential to be an effective instrument for neurodegenerative disease diagnosis and monitoring in medical contexts, improving patient care and treatment results. The absence of a control-matched group is one of the limitations in our study. Confounding effects could be introduced since the healthy controls utilised for training were not specifically matched to PD individuals on age, scanner type, or acquisition methodology. Furthermore, the significance of the observed brain age gap in the PD group was not evaluated using any formal statistical comparisons (e.g., effect size or *p*-value). These results should therefore be regarded as initial and exploratory in nature. To address this, future research will use statistical inference to robustly assess the significance and generalisability of the observed effects, as well as age- and scanner-matched controls.

## Figures and Tables

**Table 1:** Performance of SAGEFusionNet model on anatomical networks constructed using different values of the sparsity parameter λ. Bold values indicate the best performance.

Model	Sparsity Parameter	MAE	PCC
SAGEFusionNet	0.02	4.51±0.37	0.67±0.05
	0.03	4.61±0.38	0.66±0.06
	0.04	4.44±0.54	0.69±0.05
	0.05	4.48±0.39	0.67±0.07
	**0.06**	4.24±0.38	0.72±0.03
	0.07	4.29±0.38	0.70±0.04
	0.08	4.36±0.34	0.68±0.06

**Table 2:** Performance comparison of proposed and baseline models. Bold values indicate the best performance.

Model	MAE	PCC
FCNN	6.16±0.99	0.54±0.07
GCN	4.76±0.38	0.62±0.06
GraphSAGE	4.70±0.40	0.63±0.07
GAT	4.74±0.34	0.62±0.07
GIN	4.87±0.35	0.59±0.06
SAGEFusionNet	4.24±0.38	0.72±0.03

**Table 3:** Performance comparison of fusion methods. Bold values indicate the best performance.

Model	Fusion Method	Mean	PCC
SAGEFusionNet	Mean	4.60±0.45	0.65±0.07
	Max	4.52±0.51	0.66±0.06
	Sum	4.60±0.44	0.64±0.07
	Weighted Sum	4.73±0.41	0.63±0.07
	Attention	4.29±0.45	0.70±0.05
	Concatenation	4.24±0.38	0.72±0.03

**Table 4:** Performance of SAGEFusionNet with varying numbers of layers. Bold values indicate the best performance.

Model	Layers	MAE	PCC
SAGEFusionNet	2	4.34±0.42	0.70±0.05
	3	4.44±0.56	0.68±0.06
	4	4.24±0.38	0.72±0.03
	5	4.40±0.26	0.69±0.04
	6	4.56±0.39	0.65±0.06

## Data Availability

The data used in this study are publicly available from the Alzheimer’s Disease Neuroimaging Initiative (ADNI) and the Parkinson’s Progression Markers Initiative (PPMI) databases. Researchers can apply to access ADNI data at (https://adni.loni.usc.edu/, accessed on 11 January 2024) and PPMI data at (www.ppmi-info.org/access-data-specimens/download-data, accessed on 30 July 2023), RRID:SCR_006431. Access is subject to data use agreements. The code and datasets used in this study are available at: (https://github.com/NeuralLabIITGuwahati/SAGEFusionNet, accessed on 7 July 2025).
